# Phase I clinical trial of peptide vaccination with URLC10 and VEGFR1 epitope peptides in patients with advanced gastric cancer

**DOI:** 10.3892/ijo.2013.2242

**Published:** 2013-12-31

**Authors:** YOSHIE HIGASHIHARA, JUNKO KATO, AKIHITO NAGAHARA, KENTARO IZUMI, MASAE KONISHI, TOMOHIRO KODANI, NOBUKO SERIZAWA, TARO OSADA, SUMIO WATANABE

**Affiliations:** Department of Gastroenterology, Juntendo University School of Medicine, Tokyo, Japan

**Keywords:** gastric cancer, vaccination therapy, phase I clinical trial, URLC10, VEGFR1

## Abstract

Peptide vaccine treatment has attracted attention in recent years as a new therapy option for chemotherapy-resistant, advanced, unresectable cancer. The safety of peptide vaccination with HLA-A^*^2402-restricted URLC10-A24-177 and VEGFR1-A12-9 1084 epitope peptides (fixed 2-mg dose) was investigated in a phase I clinical trial of patients with advanced gastric cancer who were refractory to chemotherapy. We determined the HLA genotype of the subjects after enrollment, results of which were held by the evaluation committee and kept from both patients and investigators until completion of the study. The primary end-point was safety of the peptide vaccination. The secondary end-points were immunological responses and clinical outcome, which were compared between the HLA-A^*^2402-positive and HLA-A^*^2402-negative groups. The peptides were subcutaneously administered on day 1, 8, 15 and 22 within a 28-day treatment cycle. A total of 14 patients was enrolled in this study; 12 of the 14 patients received 4 or more vaccinations (at least 1 course). No patient had a severe treatment-related adverse event. Findings from evaluation of clinical responses after a single course showed that 4 cases had stable disease and 8 cases had progressive disease. The median overall survival time (MST) for the 12 patients was 3.9 months. The MSTs in the HLA-A^*^2402-positive and HLA-A^*^2402-negative groups were, 4.2 and 3.6 months (p= 0.9164), respectively. The results of this study showed that vaccination with URLC10 and VEGFR1 peptides was a safe treatment for advanced gastric cancer. This trial was registered with University Hospital Medical Information Network (UMIN, no. 000002409).

## Introduction

Peptide vaccine treatment has attracted attention in recent years as a new therapy option for chemotherapy resistant, advanced, unresectable cancer (1). We conducted a phase I clinical trial for gastric cancer treatment with a peptide vaccine using an URLC10 origin HLA-A^*^2402-restrictive epitope peptide and a new blood vessel antigen epitope peptide of VEGFR1. From comprehensive genomic studies, these epitopes were determined to be tumor antigens.

In recent years, combined therapy using together an anti-neoplastic drug with a molecular-targeted drug has led to an improved prognosis for chemotherapy-resistant, advanced, unresectable gastric cancer. The results of the Trastuzumab for Gastric Cancer (ToGA) Study showed that the prognosis of HER2-positive gastric cancer was improved by chemotherapy with trastuzumab, which is a human epidermal growth factor receptor (HER) 2 antibody to chemotherapy of cytotoxic agent, in combination with fluorouracil (5-FU) and CDDP (2).

However, the efficacy of existing medical treatments for advanced and recurrent gastric cancer is limited, and new more effective treatments are needed. Cancer vaccine development has advanced by the identification of various cancer-related antigens. The vaccine Sipuleucel-T (Provenge^®^) was approved as a treatment for prostate cancer by the US Food and Drug Administration (FDA) in 2010 (3). Standard treatments for gastric cancer include surgery, chemotherapy and radiotherapy; however, there has been interest recently in developing a vaccine-based therapy with fewer side-effects. Vaccine therapies are now being evaluated in clinical trials of pancreatic, esophageal, liver cell, colorectal and bile duct cancer (4–10).

Vaccination with peptides derived from vascular endothelial growth factor receptor (VEFGR) 1 has been shown to inhibit tumor growth in mice (11,12). VEGFR1 peptides established CTL clones *in vitro* from human peripheral blood mononuclear cells with HLA-A^*^2402. These CTL clones were shown to have potent cytotoxicity in an HLA class I-restricted manner not only against peptide-pulsed target cells but also against target cells endogenously expressing VEGFR1. These results strongly suggest that VEGFR1 is a promising target for an anti-angiogenic cancer vaccine.

Vaccination with upregulated lung cancer (URLC10) epitope peptide in patients with esophageal cancer was recently demonstrated to be well tolerated (13–15). Fujiwara *et al* reported that URLC10 is highly expressed in gastric cancer tissue (16).

In the present study, we employed a combination of 2 peptides: URLC10 peptide, which is highly expressed in gastric cancers, and VEGFR1 peptide. The safety of vaccination with HLA-A^*^2402-restricted URLC10 and VEGFR1 epitope-peptides was examined in patients with advanced gastric cancer refractory to chemotherapy.

## Materials and methods

### Patient eligibility

Patients diagnosed with unresectable gastric cancer refractory to chemotherapy were enrolled in this trial from August, 2009, to January, 2011, at Juntendo University Hospital, Tokyo, Japan.

### Patient inclusion criteria

The criteria for patient inclusion were as follows: unresectable/recurrent gastric cancer refractory to chemotherapy or that the treatment could not be continued because of adverse events; Eastern Cooperative Oncology Group (ECOG) performance status 0–2; age over 20 years but less than 85 years; presence or absence of measurable or evaluable lesions by Response Evaluation Criteria in Solid Tumors (RECIST) was not taken into account; surgery performed and recovery achieved or 2 weeks or more had passed since previous treatment; survival of 3 months or longer expected; white blood cell count >3,000/mm^3^ but <15,000/mm^3^, platelet count >75,000/mm^3^, aspartate aminotransferase (AST) and alanine aminotransferase (ALT) <150 IU/l, total bilirubin <3.0 mg/dl and creatinine <2.0 mg/dl; and written informed consent provided prior to the trial.

### Patient exclusion criteria

The criteria for exclusion of patients were: pregnancy or lactation; uncontrollable severe infectious diseases; receiving treatment with steroid or immunotherapy at the time of the clinical trial; presence of 2 or more uncontrollable malignancies; presence of severe trauma; insufficient recovery from an injury; and being judged inappropriate as a participant by doctors.

### Study design and treatment schedule

This study was a phase I clinical trial in patients with advanced gastric cancer that became chemotherapy resistant. The HLA-A^*^2402 restrictive epitope peptides URLC10 and VEGFR1 were prepared in incomplete Freund’s adjuvant (IFA) and injected subcutaneously, each at a dose of 2 mg, in the inguinal region or an axillary region of the patients on days 1, 8, 15 and 22 of the 28-day treatment cycle. Safety was evaluated weekly for up to 2 weeks after the last dose of medication. Patients were considered to have completed therapy if they received one or more courses. The curative outcome was analyzed approximately 29 days from the start of treatment. The administration of peptide vaccination was continued as long as possible until disease progression.

No report exists on whether there is any effect to non-HLA-A24 group vaccine so far. Therefore, patients were enrolled regardless of the HLA genotype. To assess the safety of the vaccine and to compare the efficacy without any bias, we disclosed the HLA genotypes in all cases after the completion, and the results were compared. The data were held by an evaluation committee and both patients and investigators were blinded as to the results until completion of the study. Data of the study end-points were compared between the HLA-A^*^2402-positive group and the HLA-A^*^2402-negative group.

### End-points

The primary end-point was the safety of the peptide vaccination, which was evaluated according to the National Cancer Institute Common Toxicity Criteria (Ver.3.0). Immunological reaction at the injection site (RAI) was defined by erythema and/or induration. Toxicity was evaluated 2 weeks from the last administration. The secondary end-points were immunological responses and clinical outcomes in patients who received at least one course of vaccination. Clinical outcomes included assessment by CT scanning in accordance with RECIST criteria, time to progression (TTP), and overall survival (OS). Tumor reduction was evaluated at the end of 1 course of therapy according to RECIST criteria. CT scanning was performed after the first and second cycles, and after every cycle thereafter. TTP was determined as the time from the date of the initial vaccination until the documentation of clear disease progression. OS was calculated from the date of the initial vaccination to the date of death from any cause.

### Definition of dose-limiting toxicity

Dose-limiting toxicity (DLT) was defined as hematological toxicity of grade 4 or non-hematological toxicity of grade 3 or greater (excluding nausea and vomiting) when it could not be ruled out that peptide vaccination was the cause. DLT evaluation period was until 2 weeks after the last administration.

### Peptides

VEGFR1-peptide was manufactured in accordance with Good Manufacturing Practice by the American Peptide Company Inc. (Sunnyvale, CA, USA). URLC10-derived from LY6K-177 (RYCNLEGPPI) that bound to HLA-A molecule was synthesized using metallopanstimulin (MPS).

### Statistical analysis

TTP and OS curves were estimated using Kaplan-Meier methodology.

## Results

### Patient characteristics

A total of 14 patients was enrolled in this study. We analyzed adverse events as primary end-point in all 14 cases. Two cases were excluded from secondary end-point because they were not able to complete one course of vaccination. Among them, one case found it too difficult to come to the hospital due to exacerbation of cancer pain and another case with PS 2 from the beginning, was admitted to a hospital near his home. These cases were not related to the vaccination, or due to progression of primary disease. Number of administrations of vaccine during this study was twice the minimum number of times, 44 times the maximum number of times, 11 times the average number of times. The characteristic of the 14 patients (10 males, 4 females: average age 60.3 years) are shown in Table I. ECOG PS was 0 in 6 patients, 1 in 6 patients, and 2 in 2 patients. All 14 patients had previously received chemotherapy, with 7 patients also undergoing surgery. A total of 11 patients had liver metastasis, 7 had peritoneal dissemination, 7 had lymph node metastasis, 2 had esophagus invasion, and 1 had lung metastasis. Eight of 12 patients who were able to complete the course belonged to the HLA-A^*^2402-positive group.

### Toxicity

All 14 patients were evaluated for adverse events (Table II). No patients had a severe adverse event in relation to the vaccine treatment. Five of 14 patients had RAI; i.e., erythema, induration or pruritus (Table III).

### Clinical outcomes

**Figure 1.**
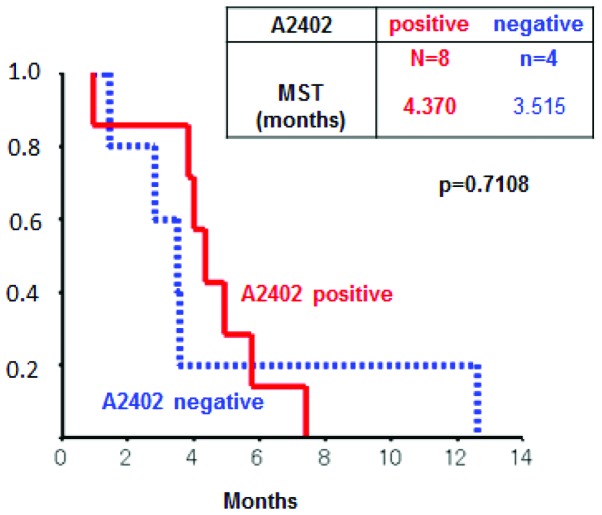
Kaplan-Meier overall survival curve. The MSTs in HLA-A^*^2402-positive group and HLA-A^*^2402-negative group were 4.2 and 3.6 months (p= 0.9164), respectively. There was no significant difference.

The evaluation after 1 course showed stable disease in 4 cases and progressive disease in 8 cases (Table III). The 3 cases with stable disease were HLA-A^*^2402-positive. The MST was 3.9 months when all 12 patients were analyzed. The MSTs in the HLA-A^*^2402-positive group and the HLA-A^*^2402-negative group were 4.2 and 3.6 months (p=0.9164), respectively (Fig. 1).

### CTL response

**Figure 2.**
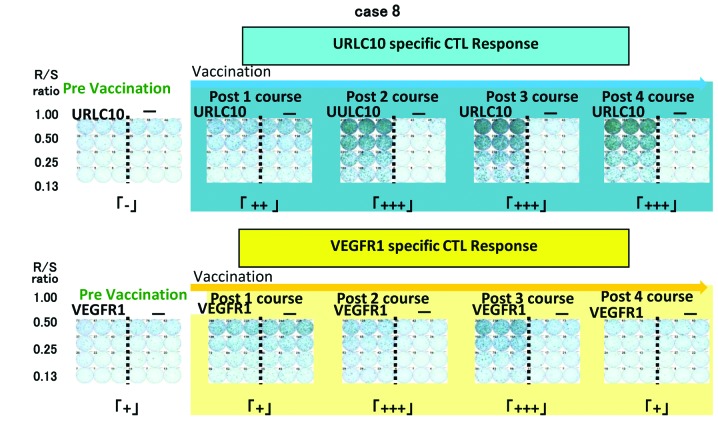
Representative immunological monitoring assays detecting antigen-specific CTL response. The cultured lymphocytes were subjected to ELISPOT assay after depletion of CD4-positive cells by magnetic beads. TISI were incubated with responder cells in the presence of URLC10 peptide or VEGFR1 peptide as irrelevant control and spot counts were quantified. CTL responses are evaluated and classified based on the algorithm [Kono *et al* (14)].

An INF-γ ELISPOT assay was conducted using peripheral blood monocytes periodically obtained from patients to assess the cellular immune responses to URLC10 and VEGFR1. Positive CTL responses specific to the vaccinated peptide were determined as previously described. Positive CTL responses were seen in 5 of 8 patients (62.5%) for URLC10 and 4 patients (50%) for VEGFR1 (Table IV, Fig. 2).

## Discussion

Tumor antigens have been identified in a variety of carcinomas, and many clinical trials have been conducted to prove the efficacy of cancer vaccinations, since the recognition of cancer-associated antigen by cytotoxic T cells (CTL) was first reported by Van der Brungeen *et al* in 1991 (17). In 2010, the US FDA subsequently approved an autologous cellular vaccine (sipuleucel-T; Provenge^®^) for the treatment of prostate cancer (3).

Clinical trials are now underway to evaluate the safety and efficacy of the vaccination for the treatment of malignant melanoma and cancers of lung, kidney, pancreas, bile duct, colon and esophagus (5,17–20). We previously reported that vaccination with KIF20A and VEGFR1 epitope peptides was safe and feasible for the treatment of patients with advanced pancreatic cancer (6). In 2010, Masuzawa *et al* reported the results of a phase I/II clinical trial in which the safety of vaccination with VEGFR1 and VEGFR2 peptide combined with S-1 and cisplatin was demonstrated in patients with advanced gastric cancer (21). However, to date, there have been no reports demonstrating the efficacy of the vaccination for the treatment of gastric cancer. In the present study, we investigated the safety of peptide vaccination with HLA-A^*^2402-restricted URLA10 and VEGFR1 epitope peptides in patients with advanced gastric cancer refractory to chemotherapy. In many of the clinical cancer vaccine trials, maximum tolerated dose (MTD) has not been observed. Many kinds of peptides which were employed in previous studies had a fixed dose (5,6,22). Further, dose escalation was not recognized in a previous colon cancer trial (23). Therefore, we did not examine dose escalation and fixed dose of peptide was used in this study. In our study, grade 3 or 4 anemia was observed in approximately 33% of the patients, and anorexia of grade 2 or more was reported for 25% of the patients, although these events were associated with progression of primary disease, and a total of 4 patients developed RAI. No severe adverse effects caused by the vaccine therapy were observed. Masuzawa *et al* (21) reported on adverse effects occurring during the first 2 cycles of the combination therapy. Grade 3 or 4 neutropenia and anemia were observed in approximately 20% of the patients, while anorexia of grade 2 or more was reported for 70% of the patients. The results were almost the same as those of the SPIRITS trial, a phase III trial of S-1 plus cisplatin for first-line treatment of advanced gastric cancer (24). A total of 6 patients developed RAI and 2 patients developed an ulcer at the injection sites. No severe adverse effects caused by the vaccine therapy were reported. In the study by Masuzawa *et al* (21), the percentage of patients with anorexia was higher than in the current study, presumably because in their study cytotoxic chemotherapy with cisplatin and S1 was combined with vaccination, and the percentages of patients with physical symptoms and blood toxicity were similar to those reported in the SPIRITS trial, indicating that vaccination can be used safely with chemotherapy. In the present study, the MST was 3.9 months when all 12 patients were analyzed. The MSTs in the HLA-A^*^2402-positive group and HLA-A^*^2402-negative group were 4.2 and 3.6 months, and these values did not differ significantly from one another (p= 0.9164), respectively. Among the 8HLA-A2402-positive patients, 3 (37.5%) had stable disease after the end of 1 treatment course. Masuzawa *et al* (21) demonstrated that the disease control rate (i.e., the percentage of patients with a partial response or stable disease) was 100% (54.5% partial response and 45.5% stable disease) after 2 cycles of combination therapy. The median time to progression was 9.6 months and the median overall survival time was 14.2 months in patients who showed a CTL response to VEGFR2 peptide. In our study, of the 8 patients who were HLA-A^*^2402-positive, 5 (62.5%) showed a CTL-positive response to URLC10 and 4 (50%) showed a CTL-positive response to VEGFR1. Two cases had an MST of 6.6 months, indicating a strong CTL response. The effectiveness of treatment in patients with strong CTL response has been suggested. Patients with advanced cancer may not be able to secure the time needed until an antitumor immune response can be obtained because of the short time between the start of treatment until disease progression. It is believed that lymphocyte immune response may be low in patients receiving intensive chemotherapy. In our study, most patients received four or more agents; thus, it is possible that the desired immune response was not achieved in all cases. There was no significant difference in MST between the HLA-A^*^2402-positive group and HLA-A^*^2402-negative group. However, in the future, the effectiveness of this therapy will be confirmed in additional cases.

In conclusion, this study demonstrates that vaccination with URLC10 and VEGFR1 epitope peptides for advanced gastric cancer can be safely performed.

## Figures and Tables

**Table I.** Patient characteristics.

Patient no.	Gender	Age	PS	Primary tumor site	HLA	Prior therapy		
						Surgery	Chemotherapy/Radiation (RT)	Metastatic sites
1	M	43	1	C	A2402	−	S-1/CDDP, CPT-11, PTX, RT	Lung, lymph node
2	F	58	0	M	A2402	−	S-1/CDDP, CPT-11/CDDP, CPT-11, PTX, DOC	Liver
3	M	69	1	M	A2402	−	S-1/CDDP, PTX, CPT-11	Liver, lymph node
4	M	57	1	M	A2402	+	S-1/CDDP, PTX	Liver, peritoneum
5	M	70	0	A	A2402	+	S-1/CDDP, PTX, CPT-11, 5-FU/MTX	Liver, lymph node, peritoneum
6	M	59	0	M	A2402	+	S-1/CDDP, CPT-11, PTX	Liver, lymph node
7	F	45	0	C	Non-A2402	−	S-1/CDDP, CPT-11, PTX	Liver, lymph node, peritoneum
8	M	53	2	C	A2402	−	S-1/CDDP, CPT-11, PTX, RT(bone)	Liver, esophagus invasion
9	M	69	0	C	A2402	+	S-1, PTX, CPT-11/CDDP, 5-FU/MTX	Liver, peritoneum
10	M	70	1	A	Non-A2402	−	By pass, S-1/CDDP, CPT-11, PTX	Peritoneum
11	F	50	1	C	Non-A2402	−	S-1/CDDP, CPT-11/PTX	Liver, lymph node, did not complete the course
12	M	78	2	M	A2402	+	S-1, CPT-11/CDDP, PTX, 5-FU/MTX, S-1/MMC	Liver, lymph node did not complete the course
13	M	56	0		Non-A2402	+	S-1, CPT-11/CDDP, PXT	Peritoneum
14	F	68	1	UM	Non-A2402	+	S-1, S-1/PTX, PTX, CPT/CDDP	Liver, peritoneum

M, male; F, female; PS, performance status; C, cardia, M, middle body; UM, under middle body; A, antrum; S-1, tegafugimeraciloteracil; CDDP, cisplatin; CPT-11, irinotecan; PTX, paclitaxel; DOC, docetaxel; 5-FU, fluorouracil; MTX, methotrexate.

**Table II.** Summary of toxicity and dermatology.

	Grade 1	Grade 2	Grade 3	Grade 4	Total patients n=14 (%)
Blood/bone marrow					
Anemia	0	2	3	2	7 (50.0)
Elevated ALP	2	0	2	1	5 (35.7)
Elevated ALT	1	0	0	0	1 (8)
Hypoalbuminemia	0	1	2	0	3 (21.4)
Creatinine	2	1	1	0	4 (33.3)
Hyperuricemia	0	0	0	2	2 (17)
Hyponatremia	0	1	0	0	1 (8)
Constitutional symptoms					
Fatigue	3	1	0	0	4 (33.3)
Anorexia	2	0	3	0	5 (35.7)
Edema	2	0	0	0	2 (17)
Nausea/vomiting	1	0	0	0	1 (8)
Diarrhea	1	0	0	0	1 (8)
Alopecia	1	0	0	0	1 (8)
Dermatology					
Rash	3	0	0	0	3 (21.4)
Induration	2	0	0	0	2 (17)
Pruritus	2	0	0	0	2 (17)

**Table III.** Clinical outcomes.

Patient no.	HLA-A^*^2402	Injection times of peptid	Course				PFS (day)	OS (day)
			1	2	3	4		
1	A2402	4th	PD				24	29
2	A2402	12th	PD				28	117
3	A2402	16th	PD				24	133
4	A2402	4th	SD	SD	PD		28	122
5	A2402	8th	PD				24	86
6	A2402	8th	SD				56	150
7	Non-A2402	12th	PD				27	109
8	A2402	19th	PD				27	175
9	A2402	16th	SD				63	226
10	Non-A2402	6th	PD				26	168
11	Non-A2402	2th					NA	
12	A2402	3th					NA	
13	Non-A2402	44th	SD	SD	SD	PD	126	384
14	Non-A2402	12th	PD				28	242

SD, stable disease; PD, progression disease; NA, not analyzed.

**Table IV.** CTL response.

Patient no.	Course	CTL response	
		URLC10 CTL response co-culture	VEGFR1 CTL response co-culture
1	Pre	+	+
	Post1	−	−
2	Pre	+	+
	Post1	−	−
	Post2	+	+
3	Pre	−	−
	Post1	−	−
	Post2	−	+
	Post3	−	−
	Post4	+	−
4	Pre	−	−
	Post1	+	+
5	Pre	+	−
	Post1	+	−
6	Pre	+	−
	Post1	−	−
	Post2	+	+
8	Pre	−	+
	Post1	++	+
	Post2	+++	+++
	Post3	+++	+++
	Post4	+++	+
9	Pre	−	+
	Post1	NA	NA
	Post2	−	+
	Post3	+++	−

NA, not analyzed.
